# N^…^C and S^…^S Interactions in Complexes, Molecules, and Transition Structures HN(CH)SX:SCO, for X = F, Cl, NC, CCH, H, and CN

**DOI:** 10.3390/molecules24183232

**Published:** 2019-09-05

**Authors:** Janet E. Del Bene, Ibon Alkorta, José Elguero

**Affiliations:** 1 Department of Chemistry, Youngstown State University, Youngstown, OH 44555, USA; 2 Instituto de Química Médica (CSIC), Juan de la Cierva, 3, E-28006 Madrid, Spain

**Keywords:** noncovalent interactions, structures, binding energies, charge-transfer energies, EOM-CCSD spin-spin coupling constants

## Abstract

Ab initio Møller–Plesset perturbation theory (MP2)/aug’-cc-pVTZ calculations have been carried out in search of complexes, molecules, and transition structures on HN(CH)SX:SCO potential energy surfaces for X = F, Cl, NC, CCH, H, and CN. Equilibrium complexes on these surfaces have C_1_ symmetry, but these have binding energies that are no more than 0.5 kJ·mol^–1^ greater than the corresponding C_s_ complexes which are vibrationally averaged equilibrium complexes. The binding energies of these span a narrow range and are independent of the N–C distance across the tetrel bond, but they exhibit a second-order dependence on the S–S distance across the chalcogen bond. Charge-transfer interactions stabilize all of these complexes. Only the potential energy surfaces HN(CH)SF:SCO and HN(CH)SCl:SCO have bound molecules that have short covalent N–C bonds and significantly shorter S**^…^**S chalcogen bonds compared to the complexes. Equation-of-motion coupled cluster singles and doubles (EOM-CCSD) spin-spin coupling constants ^1t^J(N–C) for the HN(CH)SX:SCO complexes are small and exhibit no dependence on the N–C distance, while ^1c^J(S–S) exhibit a second-order dependence on the S–S distance, increasing as the S–S distance decreases. Coupling constants ^1t^J(N–C) and ^1c^J(S–S) as a function of the N–C and S–S distances, respectively, in HN(CH)SF:SCO and HN(CH)SCl:SCO increase in the transition structures and then decrease in the molecules. These changes reflect the changing nature of the N**^…^**C and S**^…^**S bonds in these two systems.

## 1. Introduction

The field of intermolecular interactions has expanded dramatically from the hydrogen bond described in detail in Pimentel’s classic book [1], to other types of intermolecular interactions that occur as an electron-pair in one molecule is donated to another molecule through its σ- or π-hole. These bonds have been named by the group in the Periodic Table that contains the electron-acceptor atom. Thus, intermolecular interactions include halogen bonds involving group 17 atoms as the acids [2,3,4], chalcogen bonds for group 16 [5,6,7,8], pnicogen bonds for group 15 [9,10,11], and tetrel bonds for group 14 [12,13,14]. Legon and Resnati, et al. have emphasized the similarities among these bonds and suggested that they should be considered as arising when a σ-hole [15,16] or a π-hole associated with an E atom in one molecular entity interacts with a nucleophilic region such as a pair of nonbonding or π electrons in another, or the same, molecular entity [17].

**Scheme 1:**
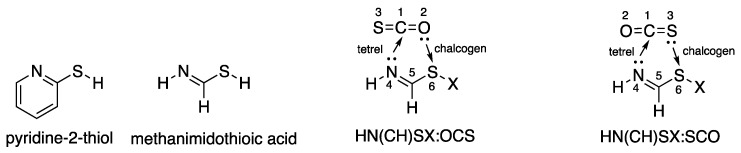
Monomers and complexes formed from HN(CH)SX and S=C=O.

Our continuing interest in the types of noncovalent bonds described above has led us to systematically explore intermolecular bonds that involve O=C=O and O=C=S. We have examined tetrel bond formation involving O=C=O with a series of bases, as well as tetrel and chalcogen bond formation involving HN(CH)SX and O=C=O, for a series of substituents X [18,19,20,21,22]. These studies have led us to investigate a related series of complexes involving O=C=S and nitrogen bases, and subsequently O=C=S and HN(CH)SX in complexes stabilized by N**^…^**C tetrel bonds and O**^…^**S chalcogen bonds [23,24]. The molecule HN(CH)SX is quite interesting as a simple model of pyridine-2-thiol (shown in Scheme 1) and 2-(Xthio)pyridines, compounds that have useful chemical and pharmacological properties [25,26]. The formation of tetrel and chalcogen bonds involving HN(CH)SX with S=C=O is illustrated in Scheme 1.

In the present study, we have extended our investigation of the interaction of HN(CH)SX with O=C=S in order to examine N**^…^**C tetrel bonds and S**^…^**S chalcogen bonds, as illustrated in Scheme 1. The HN(CH)SX:OCS complex shown in Scheme 1 could be described as a “trans” complex to indicate that the C–S bond of O=C=S is trans to the N4–C5 bond of the HN(CH)SX molecule with respect to the C1–N4 bond. In such complexes, the chalcogen bond is an O**^…^**S bond. In the HN(CH)SX:SCO complexes, these same two bonds have a cis orientation. For ease of discussion, we refer to the “cis” complexes as HN(CH)SX:SCO to emphasize the presence of an S**^…^**S chalcogen bond. The “trans” complexes with which they are compared are written as HN(CH)SX:OCS to indicate the presence of an O**^…^**S chalcogen bond. In this paper, we present the equilibrium complexes and molecules found on the HN(CH)SX:SCO potential energy surfaces, as well as the transition structures that interconvert the complexes and molecules. We discuss the structures and binding energies of these complexes, molecules, and transition structures; selected properties including charge-transfer energies in the complexes; and spin-spin coupling constants across tetrel and chalcogen bonds for complexes, molecules, and transition structures. Finally, we compare the properties of the HN(CH)SX:SCO complexes, molecules, and transition structures with those of the isomers HN(CH)SX:OCS [24].

## 2. Methods

The structures of HN(CH)SX:SCO complexes and molecules for X = F, Cl, NC, CCH, H, and CN that are stabilized by N**^…^**C tetrel bonds and S**^…^**S chalcogen bonds were optimized at second-order Møller–Plesset perturbation theory (MP2) [27,28,29,30] and the aug’-cc-pVTZ basis set [31]. This basis set was derived from the Dunning aug-cc-pVTZ basis set [32,33] by removing diffuse functions from H atoms. No basis set superposition error correction has been made because it has been demonstrated that the aug’-cc-pVTZ basis set reduces the basis set superposition error, and aug’-cc-pVXZ basis sets have improved convergence properties compared to the full aug-cc-pVXZ basis sets [31]. Transition structures that interconvert equilibrium complexes and molecules were also optimized. Frequencies were computed to establish that the equilibrium structures have no imaginary frequencies and that the transition structures have one imaginary frequency along the coordinate that interconverts the two equilibrium structures. Optimization and frequency calculations were performed using the Gaussian 16 program [34]. The binding energies of the binary complexes, molecules, and transition structures were computed as (–ΔE) for the reaction that forms these moieties from the corresponding isolated monomers.

The natural bond orbital (NBO) method [35] has been used to obtain the stabilizing charge-transfer interactions in complexes using the NBO-6 program [36]. The charge-transfer interactions were computed using the B3LYP functional with the aug’-cc-pVTZ basis set at the MP2/aug’-cc-pVTZ complex geometries so that at least some electron correlation effects could be included. The atoms in molecules (AIM) methodology [37,38,39,40] was used to produce the molecular graphs of complexes, molecules, and transition structures employing the AIMAll [41] program. The molecular graph identifies the location of electron density features of interest, including the electron density (ρ) maxima associated with the various nuclei and saddle points which correspond to bond critical points (BCPs). The zero gradient line which connects a BCP with two nuclei is the bond path.

Coupling constants were evaluated using the equation-of-motion coupled cluster singles and doubles (EOM-CCSD) method in the CI (configuration interaction)-like approximation [42,43] with all electrons correlated. For these calculations, the Ahlrichs [44] qzp basis set was placed on ^13^C, ^15^N, ^17^O, and ^19^F, the qz2p basis set on ^33^S and ^35^Cl, and the Dunning cc-pVDZ basis on ^1^H atoms. All terms, namely, the paramagnetic spin orbit (PSO), diamagnetic spin orbit (DSO), Fermi contact (FC), and spin dipole (SD) were evaluated. The EOM-CCSD calculations were performed using ACES II [45] on the HPC cluster Owens at the Ohio Supercomputer Center.

## 3. Results and Discussion

The results obtained in this study are presented below in five sections. Section 1 addresses the equilibrium HN(CH)SX:SCO complexes that have C_1_ symmetry. In the second section, corresponding complexes that have C_s_ symmetry are discussed. In Section 3, the molecules found on the potential surfaces, and the transition structures that interconvert complexes and molecules are presented. Section 4 presents spin-spin coupling constants for complexes, molecules, and transition structures. In the fifth section, some comparisons are made between HN(CH)SX:SCO complexes that are stabilized by N**^…^**C tetrel and S**^…^**S chalcogen bonds, and HN(CH)SX:OCS complexes that have N**^…^**C tetrel and O**^…^**S chalcogen bonds.

### 3.1. HN(CH)SX:SCO Complexes with C_1_ Symmetry

**Figure 1:**
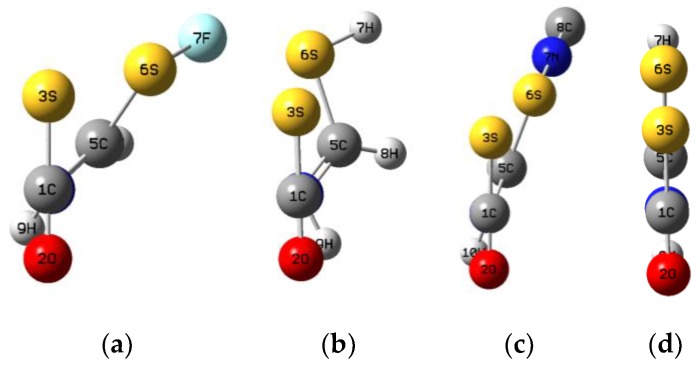
HN(CH)SX:SCO complexes with X = (**a**) F, *C*_1_; (**b**) H, *C*_1_; (**c**) NC, *C*_1_; and (**d**) H, *C_s_*.

Searches of the HN(CH)SX:SCO potential energy surfaces yielded equilibrium complexes containing N**^…^**C tetrel and S**^…^**S chalcogen bonds. The structures, total energies, and molecular graphs of these complexes are given in Table S1 of Supporting Information. In the molecular graphs, these complexes appear to have planar *C_s_* symmetry, but they do not. Rather, they have *C*_1_ symmetry, as illustrated in Figure 1 for complexes with X = F, NC, and H. In this figure, the S=C=O molecule has been oriented so that it is perpendicular to the N**^…^**C tetrel bond, and the view is along that bond. This orientation illustrates the deviation of these complexes from *C_s_* symmetry. The complexes with X = F, Cl, CCH, H, and CN have dihedral S–C–N–C angles between 39 and 49°, and they are represented by HN(CH)SF:SCO and HN(CH)SH:SCO in Figure 1a,b, respectively. The dihedral angle is reduced to 20° in the complex with X = NC, which is illustrated in Figure 1c. The planar HN(CH)SH:SCO *C_s_* complex is shown in Figure 1d. Figure 1 also provides insight into the values of the S–S–A angles in these complexes. Complexes X = F, Cl, and NC have values of this angle between 166 and 169°. These values are consistent with a traditional S**^…^**S chalcogen bond, in which a lone pair on S3 is directed toward the σ-hole on S6. Complexes with X = CCH, H, and CN have S–S–A angles of 123, 131, and 124°, respectively, which is not an optimum orientation for the formation of an S**^…^**S chalcogen bond.

Table 1 provides structural and energetic data for complexes with *C*_1_ symmetry, including binding energies; N–C and S–S distances across the tetrel and chalcogen bonds, respectively; the N–C–S and S–S–A angles; and the dihedral S–C–N–C angles. In the tables, atoms are given numbers that correspond to Figure 1, but in the text, numbers are not used unless there might be some ambiguity. The binding energies of these complexes span a small range from 13.3 to 15.6 kJ·mol^–1^, and they decrease with respect to X in the order F > Cl > NC = CCH > H ≈ CN. Thus, the complexes with the stronger electron-withdrawing substituents have the greater binding energies.

Further insight into these complexes can be gained by examining their S–S and N–C distances. The three most strongly bound complexes have S–S distances between 3.10 and 3.50 Å, with the most strongly bound complex having the shortest S–S distance. As noted previously, these three complexes have S–S–A angles of 169, 166, and 166°, respectively, values consistent with a chalcogen bond. In contrast, the S–S distance is much longer in the complexes with X = CCH, H, and CN, varying between 3.78 and 3.81 Å. These complexes also have reduced S–S–A angles of 123, 131, and 124°, respectively. These data indicate that the chalcogen bonds in the latter complexes are very weak or perhaps nonexistent.

The most strongly bound complex with X = F has an N–C distance of 3.01 Å. The N–C distances in the complexes with X = Cl and NC are 3.06 Å. This distance in the remaining complexes with X = CCH, H, and CN is shorter, with values between 3.02 and 3.04 Å. These short N–C distances suggest that in the complexes with the weaker S**^…^**S chalcogen bonds, the N**^…^**C tetrel bonds may be stronger. The values of the N–C–S angle are consistent with the formation of a tetrel bond at C through its local π-hole.

**Figure 2:**
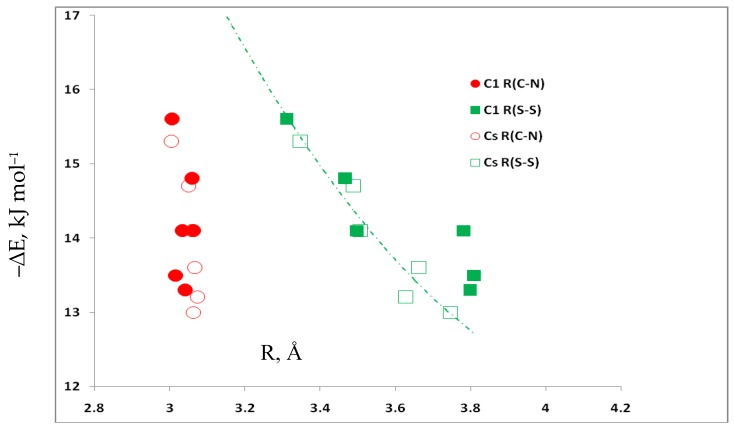
Binding energies of *C*_1_ and *C_s_* HN(CH)SX:SCO complexes vs. the N4–C1 and S–S distances, respectively.

Figure 2 provides plots of the binding energies of the HN(CH)SX:SCO complexes versus the N–C and S–S distances. It is evident from this figure that the binding energies are essentially independent of the N–C distance. Moreover, there is not a good correlation between these binding energies and the S–S distance. This lack of correlation may be attributed to the differences primarily in the S**^…^**S bonds in complexes with X = F, Cl, and NC compared to those having X = CCH, H, and CN.

The nature of the charge-transfer interactions and their energies for the *C*_1_ complexes are reported in Table 2. There are two lone pairs of electrons on S3, and each can be involved in charge-transfer. The strongest S3_lp1_ → σ*S6–A interactions are found for the complexes with X = F, Cl, and NC, with values of 12.2, 8.0, and 5.9 kJ**^.^**mol^–1^, respectively. The charge-transfer energies drop to less than 1 kJ**^.^**mol^–1^ for the complexes with X = CCH, H, and CN. The second charge-transfer interaction has a value of about 2 kJ**^.^**mol^–1^ for the complexes with X = F, Cl, and NC, and it is less than 0.1 kJ**^.^**mol^–1^ for the complexes with X = CCH, H, and CN. Thus, these data for the S**^…^**S bonds are consistent with the previous statements that chalcogen bonds in complexes with X = CCH, H, and CN are very weak or nonexistent.

There are also two charge-transfer interactions across the tetrel bonds. The dominant interaction arises from the donation of the lone pair on N to the local in-plane antibonding π C–O orbital. The charge-transfer energies range from 2.5 to 5.9 kJ·mol^–1^ and decrease in the order F > Cl = NC > H > CCH ≈ CN. There is also a second charge-transfer in the complexes with X = F, Cl, NC, and H arising from electron donation from the lone pair on N to the local π*C–S orbital, with energies of only 0.3 kJ·mol^–1^.

### 3.2. HN(CH)SX:SCO Complexes with C_s_ Symmetry

**Figure 3:**
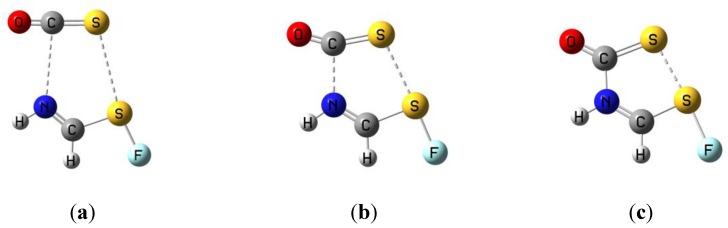
HN(CH)SF:SCO (**a**) complex; (**b**) transition structure; and (**c**) molecule with *C_s_* symmetry.

The structures, total energies, and molecular graphs of the optimized HN(CH)SX:SCO complexes with *C***_s_** symmetry are reported in Table S2 of Supporting Information. Table 3 reports their binding energies, N–C and S–S distances, and N–C–S and S–S–A angles, and Figure 3a depicts the planar HN(CH)SF:SCO complex. The *C*_s_ complexes have one imaginary frequency and are thus the transition structures that connect the two equivalent *C*_1_ complexes. However, the difference between the binding energies of corresponding *C_s_* and *C*_1_ structures does not exceed 0.5 kJ**^.^**mol^–1^, found for the complexes with X = CCH and H. When X = NC, the binding energies of the *C*_1_ and *C_s_* structures are the same. From these data, it is apparent that the potential energy surfaces are very flat in the region surrounding the equilibrium and transition structures. Thus, the *C_s_* structures may be viewed as vibrationally averaged structures, and these structures will be discussed below in this and the following sections.

Figure 2 also provides plots of the binding energies of the complexes with *C_s_* symmetry versus the N–C and S–S distances. Once again, it is apparent that these binding energies are essentially independent of the N–C distance, but they do depend on the S–S distance. The correlation coefficient of the second-order trend line for the complexes with *C_s_* symmetry is 0.922. This plot also shows the similarities between the binding energies and S–S distances for the *C*_1_ and *C_s_* complexes when X = F, Cl, and NC, as well as the relatively small but noticeable differences when X = CCH, H, and CN.

It is interesting to compare the corresponding N–C and S–S distances across tetrel and chalcogen bonds, respectively, in the *C*_1_ and *C_s_* complexes. The N–C distances in the *C_s_* complexes with X = F, Cl, and NC are slightly shorter than in the corresponding *C*_1_ complexes, but the difference does not exceed 0.008 Å. In contrast, these distances are longer by 0.03–0.05 Å in the *C_s_* complexes with X = CCH, H, and CN. For the complexes with X = F, Cl, and NC, the S–S distances in the *C_s_* structures are longer by 0.01–0.04 Å compared to the corresponding *C*_1_ complexes, but this distance is shorter in the *C*_1_ complexes with X = CCH, H, and CN by 0.06–0.17 Å. The N4–C1–S3 angles in all of the complexes are consistent with tetrel-bond formation through the π-hole at C of S=C=O. The S–S–A angles are indicative of S**^…^**S chalcogen-bond formation in the all of the *C_s_* complexes and in the *C*_1_ complexes with X = F, Cl, and NC through the σ-hole on S6, but indicate that this bond is distorted when X = CCH, H, and CN.

Table 2 reports the charge-transfer energies for the *C_s_* complexes. The dominant charge-transfer interaction across the tetrel bond arises from electron donation by N to the local in-plane antibonding π C–O orbital. The charge-transfer energy is 6.6 kJ**^.^**mol^–1^ for the complex with X = F which has an N–C distance of 3.004 Å, and 5.4 kJ**^.^**mol^–1^ at an N–C distance of 3.051 Å when X = Cl. In the remaining complexes, the charge-transfer energies are between 4.8 and 5.2 kJ**^.^**mol^−1^ at N–C distances between 3.060 and 3.075 Å. There is also a second charge-transfer of about 0.3 kJ**^.^**mol^–1^ across the tetrel bond from the lone pair on N to the local in-plane π antibonding C–S orbital.

There are two lone pairs of electrons on S3, and there are therefore two charge-transfer interactions across the S3**^…^**S6 chalcogen bonds. The primary charge transfer arises from donation of one of the lone pairs on S3 to a σ antibonding S–A orbital. These charge-transfer energies vary from 3.5 to 12.8 kJ**^.^**mol^–1^ and decrease with respect to X in the same order as the binding energies. A plot of these energies versus the S–S distance has a second-order trend line with a correlation coefficient of 0.935. It is noteworthy that all of these energies are greater than the charge-transfer energies in the corresponding *C*_1_ complexes, particularly for the complexes with X = CCH, H, and CN. The second charge-transfer interaction across the chalcogen bond is much weaker, with values between 0.4 and 1.7 kJ**^.^**mol^–1^.

### 3.3. HN(CH)SX:SCO Molecules and Transition Structures

Searches of the potential energy surfaces led to the identification of HN(CH)SX:SCO transition structures and molecules, which are illustrated by HN(CH)SF:OCS in Figure 3b,c, respectively. The structures, total energies, and molecular graphs of the molecules and transition structures are given in Tables S3 and S4, respectively, in Supporting Information. The binding energies, N–C and S–S distances, and N–C–S and S–S–A angles of the molecules are reported in Table 4. The molecules have a significantly reduced N–C covalent bond distance of 1.44 Å when X = F, Cl, and NC. In the complexes with X = CCH, H, and CN, this distance is longer, varying between 1.52 and 1.55 Å. The S–S distances are also much shorter in the molecules than they are in the complexes, with a value of approximately 2.30 Å for the molecules with X = F, Cl, and NC, and values are between 2.67 and 2.89 Å in the remaining molecules. The reduced values of the S–S distance indicate that the chalcogen bonds have acquired increased covalent character in the molecules relative to the complexes. The data also suggest that the N–C covalent bond and the S**^…^**S chalcogen bond are significantly weaker in the molecules when the substituents are CCH, H, and CN.

The data of Table 4 illustrate that the distance changes are also accompanied by angular changes in the molecular geometries. The N4–C1–S3 angle increases dramatically from about 96° in the complexes to between 131 and 137° in the molecules. This is as expected as the N**^…^**C tetrel bond becomes an N–C covalent bond. Except for HN(CH)SH:SCO, the S–S–A angle increases relative to its value in the complexes and approaches 180° in the molecules, the idealized value for a chalcogen bond.

**Figure 4:**
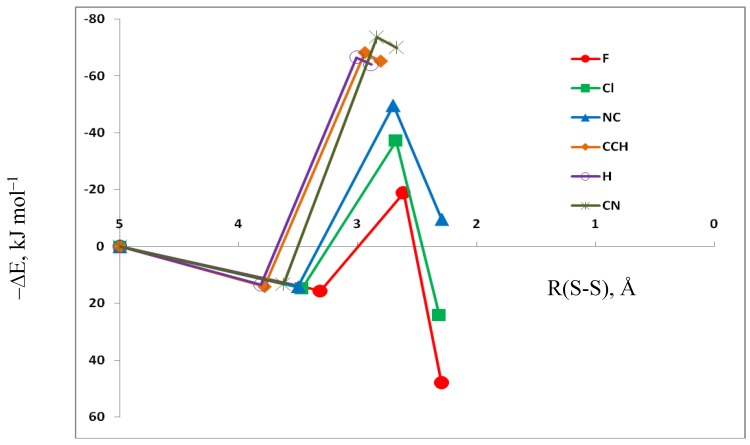
Binding energies of complexes, transition structures, and molecules as a function of the S–S distance. The points at 5 Å represent the noninteracting monomers HN(CH)SX and S=C=O.

The binding energies of the molecules are also reported in Table 4. Only the molecules HN(CH)SF:SCO and HN(CH)SCl:SCO are bound relative to the corresponding isolated monomers, with binding energies of 48 and 24 kJ**^.^**mol^–1^, respectively. HN(CH)S(NC):SCO is unbound but by only −10 kJ·mol^–1^. The remaining molecules with X = CCH, H, and CN are unbound by between −64 and −70 kJ·mol^–1^. Thus, the covalent N–C bond and the S**^…^**S chalcogen bond are sufficiently strong to overcome the distortion energies of the monomers only when X = F and Cl. Figure 4 illustrates the stabilization energies of complexes, transition structures, and molecules as a function of the S–S distance. A similar plot can be obtained as a function of the N–C distance.

It is informative to examine the changes that occur in the geometries of HN(CH)SF:SCO and HN(CH)SCl:SCO as these complexes traverse the transition states and become molecules. For ease of comparison, Table 5 presents the N–C, S–S, and S–A distances, as well as the N–C–S, S–S–A, and S–C–O angles for the complexes, transition structures, and molecules. As the two complexes become molecules, the N**^…^**C tetrel bond becomes an N–C covalent bond. Thus, the N–C distance decreases dramatically from about 3.0 Å to 1.44 Å, the N–C–S angle increases from 95 to 109°, and the S=C=O molecule becomes nonlinear as the S–C–O angle decreases to 131°. The values of these geometric descriptors in the transition structure lie between the values found in the complex and corresponding molecule, except for the S–S–A angle, which is similar in the molecule and transition structure. Significant changes are also found for the S**^…^**S chalcogen bond. In particular, the S–S distance decreases dramatically from 3.35 and 3.49 Å in the complexes with X = F and Cl to 2.29 and 2.31 Å, respectively, in the molecules as the S–S–A angle increases from 166 to about 176°. It is noteworthy that the S–F distance increases from 1.64 to 1.80 Å, while the S–Cl distance increases from 2.04 to 2.31 Å as the complexes become molecules. This suggests that the formation of the molecule leads to a weakening of the S–A bond.

The binding energies of the HN(CH)SF:SCO and HN(CH)SCl:SCO complexes, transition structures, and molecules are also reported in Table 5. The binding energies increase to 48 and 24 kJ·mol^–1^ for the molecules with X = F and Cl, respectively. The corresponding transition structures are unbound by −19 and −37 kJ·mol^–1^. Thus, the barrier to convert the HN(CH)SF:SCO complex to the molecule is 34 kJ·mol^–1^, while the barrier for the reverse reaction is 67 kJ·mol^–1^. For HN(CH)SCl:SCO, the barrier to convert the complex to the molecule is 52 kJ·mol^–1^, while the barrier for the reverse reaction is 61 kJ·mol^–1^. These barriers are visible in Figure 4. These data suggest that at low temperatures, the complexes would form as the two monomers approach each other. However, at higher temperatures, the HN(CH)SF:SCO molecule would be the dominant species, while both the HN(CH)SCl:SCO complex and molecule would have appreciable concentrations.

### 3.4. Spin-Spin Coupling Constants

Spin-spin coupling constants ^1t^J(N–C) across the tetrel bonds and ^1c^J(S–S) across the chalcogen bonds have been computed for all of the complexes having *C_s_* symmetry. For the transition structures HN(CH)SF:SCO and HN(CH)SCl:SCO coupling constants ^1t^J(N–C) across the tetrel bonds and ^1c^J(S–S) across the chalcogen bonds were evaluated. Coupling constants ^1c^J(S–S) were computed for the molecules as well as ^1^J(N–C) for coupling across the covalent N–C bond. The components of these coupling constants are reported in Table S5 of Supporting Information. As is usually the case, coupling constants ^1t^J(N–C) across the tetrel bond and ^1c^J(S–S) across the chalcogen bond for complexes and transition structures are determined by the FC term. This is not generally the case for molecules due to contributions from the PSO and SD terms.

**Figure 5:**
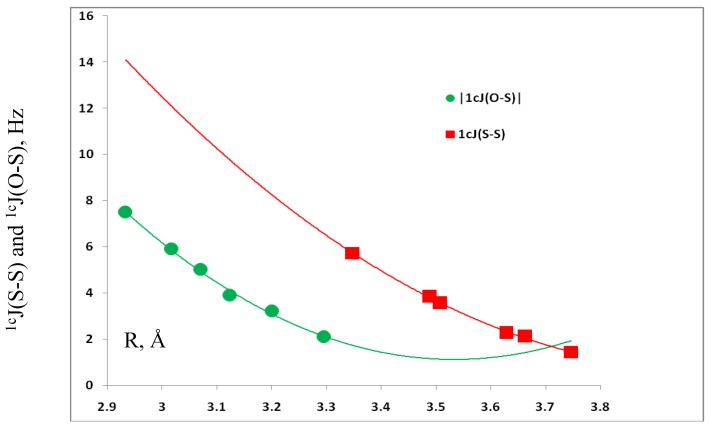
Coupling constants ^1c^J(S–S) for the HN(CH)SX:SCO complexes and the absolute values of ^1c^J(O–S) for the HN(CH)SX:OCS complexes versus the S–S and O–S distances, respectively.

Table S5 provides values of the coupling constants ^1t^J(N–C) across the tetrel bonds and ^1c^J(S–S) across the chalcogen bonds for all HN(CH)SX:SCO complexes. The ^1t^J(N–C) values are very small at −0.3 Hz in all complexes. In contrast, ^1c^J(S–S) values, which are reported in Table 3, vary from 1.4 to 5.7 Hz and decrease with respect to the substituent X in the order F > Cl ≈ NC > CN ≈ CCH > H. Figure 5 illustrates the strong dependence of this coupling constant on the S–S distance, with a correlation coefficient of 0.999 for a second-order trend line.

**Figure 6:**
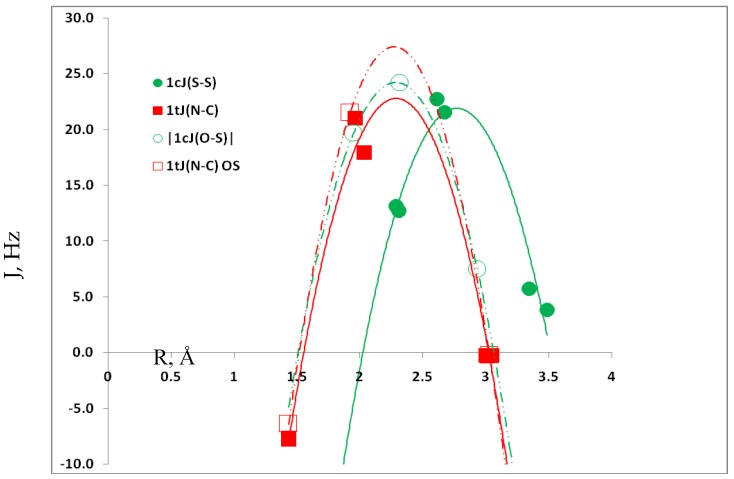
Coupling constants ^1c^J(S–S) for HN(CH)SF:SCO and HN(CH)SCl:SCO versus the S–S distance, ^1c^J(O–S) versus the O–S distance for HN(CH)SF:OCS, and ^1t^J(N–C) versus the N–C distance for corresponding complexes, transition structures, and molecules. Solid symbols refer to HN(CH)SF:SCO and HN(CH)SCl:SCO, and open symbols refer to HN(CH)SF:OCS.

The values of ^1t^J(N–C) and ^1c^J(S–S) for the HN(CH)SF:SCO and HN(CH)SCl:SCO complexes and transition structures, as well as the values of ^1c^J(S–S) and ^1^J(N–C) for the corresponding molecules, are reported in Table 6. As the N–C distance decreases in going from the complex to the transition structure, ^1t^J(N–C) increases to 18 and 21 Hz in transition structures with X = F and Cl, respectively. In the molecules, the N–C bond becomes a covalent bond, and ^1^J(N–C) decreases dramatically to −7.8 and −7.6 Hz, respectively, when X = F and Cl. Figure 6 illustrates these changes as a function of the N–C distance. The correlation coefficient of the second-order trend line is 0.980.

A similar pattern can be observed for ^1c^J(S–S) as a function of the S–S distance. This coupling constant increases from 5.7 and 3.8 Hz in the complexes to 22.7 and 21.5 Hz in the transition structures with X = F and Cl, respectively, as the S–S distance decreases. A further decrease in the S–S distance leads to a decrease in these two coupling constants to 13.1 and 12.7 Hz, respectively, but these two coupling constants do not change sign. The S···S bond gains covalent character in the transition structures and again in the molecules, but it remains an intermolecular chalcogen bond. The correlation coefficient is 0.939 for the second-order trend line in Figure 6, which illustrates these changes.

### 3.5. Comparison of C_s_ Complexes, Molecules, and Transition Structures HN(CH)SX:SCO and HN(CH)SX:OCS

Data for the isomers HN(CH)SX:OCS with the same set of substituents X but which are stabilized by O**^…^**S chalcogen bonds instead of S**^…^**S bonds have been reported in Reference [**Error! Bookmark not defined.**]. The equilibrium HN(CH)SX:OCS complexes have *C*_s_ symmetry. The binding energies of HN(CH)SX:OCS and HN(CH)SX:SCO isomers with *C*_s_ symmetry are similar. Those with O**^…^**S chalcogen bonds have binding energies between 12.2 and 15.1 kJ**^.^**mol^–1^, while those with S**^…^**S chalcogen bonds have binding energies between 13.0 and 15.3 kJ**^.^**mol^–1^. In both series, the most strongly bound complexes have X = F, Cl, and NC. The C1–N4 distances in these two series are similar, with values of 3.03–3.09 Å in the complexes with O**^…^**S chalcogen bonds and 3.00–3.08 Å in complexes with S**^…^**S bonds. The O–S distance in the complexes with O**^…^**S chalcogen bonds varies by 0.363 Å, while the S–S distance varies by 0.399 Å in complexes with S**^…^**S chalcogen bonds. In both series of complexes, the binding energies are essentially independent of the N–C distance but exhibit a second-order dependence on the O–S and S–S distances.

There is only one HN(CH)SX:OCS potential energy surface that has a bound molecule, namely HN(CH)SF:OCS. The binding energy of this molecule is 15.2 kJ·mol^–1^ at the N–C and O–S distances of 1.431 and 1.948 Å, respectively. By comparison, there are bound molecules on two HN(CH)SX:SCO potential energy surfaces, those with X = F and Cl. The binding energies of these are 48.1 and 24.3 kJ·mol^–1^, respectively, at an N–C distance of 1.44 Å and an S–S distance of approximately 2.30 Å. Thus, the two bound molecules with X = F and Cl that are stabilized by S**^…^**S chalcogen bonds are significantly more stable than the HN(CH)SF:OCS molecule with an O**^…^**S chalcogen bond.

It is also possible to compare the changes in the coupling constants ^1t^J(C–N) and ^1c^J(S–S) for HN(CH)SX:SCO complexes with the changes in ^1t^J(C–N) and ^1c^J(O–S) for HN(CH)SX:OCS. In both series, ^1t^J(N–C) is independent of the nature of the substituent and the N–C distance. In contrast, the second-order dependence of ^1c^J(O–S) and ^1c^J(S–S) on the O–S and S–S distances, respectively, is illustrated in Figure 5. The correlation coefficients are greater than 0.990. This type of distance dependence across intermolecular bonds in a related series of complexes is common when the nature of this bond does not change significantly as the bond distance changes [20,22].

The effect of the changing nature of the O**^…^**S bond in HN(CH)SF:OCS, the S**^…^**S bonds in HN(CH)SF:SCO and HN(CH)SCl:SCO, and the N**^…^**C bonds in these three complexes on C1–N4, O–S and S–S coupling constants is dramatic, as is illustrated in Figure 6. ^1t^J(N–C) increases in going from the complex to the transition structure as the N–C distance decreases, and then it decreases and changes sign in the molecule as a covalent N–C bond is formed. ^1c^J(S–S) and ^1c^J(O–S) show similar patterns as functions of the S–S and O–S distances, respectively, although these coupling constants do not change sign because the S**^…^**S and O**^…^**S bonds remain chalcogen bonds but with increased covalent character. In Figure 6, the curve for ^1c^J(S–S) is displaced to longer distances simply because the S atom has a larger van der Waals radius than O.

## 4. Conclusions

Ab initio MP2/aug’-cc-pVTZ calculations have been carried out in search of equilibrium complexes and molecules, as well as the transition structures that interconvert them on the HN(CH)SX:SCO potential energy surfaces, for X = F, Cl, NC, CCH, H, and CN. The results of these calculations support the following statements.

1. The equilibrium complexes on the HN(CH)SX:SCO surfaces have *C*_1_ symmetry, but these have binding energies that are no more than 0.5 kJ·mol^–1^ greater than the corresponding *C*_s_ complexes, which are the transition structures separating two *C*_1_ complexes. The flatness of the potential surfaces in the region of these complexes suggests that the *C*_s_ structures are vibrationally averaged equilibrium complexes. The binding energies of corresponding *C*_1_ and *C*_s_ complexes differ by no more than 0.5 kJ·mol^–1^. The following statements refer to the complexes with *C*_s_ symmetry.

2. The binding energies of the HN(CH)SX:SCO complexes span a narrow range from 13.2 to 15.3 kJ·mol^–1^. They are independent of the N–C distance across the tetrel bond but exhibit a second-order dependence on the S–S distance across the chalcogen bond.

3. Charge-transfer interactions stabilize all of these complexes. They arise primary from electron donation of the lone pair on N to the local in-plane antibonding π orbital of S=C=O, and from donation of the lone pair on S of S=C=O to the antibonding σ S–A orbital of HN(CH)SX.

4. Only the potential surfaces with X = F and Cl have bound HN(CH)SX:SCO molecules, with binding energies of 48.1 and 24.3 kJ·mol^–1^, respectively. These molecules have short covalent N–C bonds and significantly shorter S–S chalcogen bonds compared to the complexes. The barriers for converting the complexes to the molecules are 34 and 52 kJ·mol^–1^ for X = F and Cl, respectively. The barriers for the reverse reactions are 67 and 61 kJ·mol^–1^, respectively.

5. EOM-CCSD spin-spin coupling constants ^1t^J(N–C) for the HN(CH)SX:SCO complexes have a very small value of −0.3 Hz and are independent of the N–C distance. In contrast, ^1c^J(S–S) values exhibit a second-order dependence on the S–S distance, increasing as this distance decreases.

6. For complexes with X = F and Cl, spin-spin coupling constants ^1t^J(N–C) and ^1c^J(S–S) as functions of the N–C and S–S distances, respectively, increase in going from the complex to the transition structure and then decrease in the molecules. These changes reflect the changing nature of the N**^…^**C and S**^…^**S bonds. At the shorter distances in the transition structures, the N**^…^**C bond gains covalency, and then becomes a covalent N–C bond in the molecule. The S**^…^**S bond has increased covalent character in the transition structure and even more so in the molecule, but it remains a chalcogen bond.

## Figures and Tables

**Table 1:** Binding energies (–ΔE, kJ**^.^**mol^–1^), N–C and S–S distances (R, Å), N–C–S and S–S–A angles (<, ^o^), and dihedral angles S–C–N–C (dih<, °) for *C*_1_ HN(CH)SX:SCO complexes.

X =	–ΔE	R(N4–C1)	R(S3–S6)	<N4–C1–S3	<S3–S6–A7 ^a^	dih<S3–C1–N4–C5
F	15.6	3.006	3.310	93	169	46
Cl	14.8	3.059	3.467	95	166	49
NC	14.1	3.064	3.498	96	166	20
CCH	14.1	3.034	3.782	97	123	43
H	13.5	3.016	3.810	97	131	39
CN	13.3	3.041	3.798	98	124	44

^a^ A is the X atom directly bonded to S6.

**Table 2:** Charge-transfer energies (CT, kJ**^.^**mol^–1^) for *C*_1_ and *C_s_* HN(CH)SX:SCO complexes. ^a^

X =	S3_lp1_ → σ*S6–A	S3_lp2_ → σ*S6–A	N_lp_ → π*C–O	N_lp_ → π*C–S3
F	12.2 (12.8)	1.9 (1.7)	5.9 (6.6)	0.3 (0.3)
Cl	8.0 (9.5)	1.5 (0.8)	4.9 (5.4)	0.3 (0.3)
NC	5.9 (7.5)	1.6 (1.0)	4.9 (5.0)	0.3 (0.3)
CCH	0.5 (4.7)	---^b^ (0.5)	2.6 (4.8)	---^b^ (0.3)
H	0.9 (4.0)	---^b^ (0.4)	3.6 (5.1)	0.3 (0.3)
CN	0.5 (3.5)	---^b^ (0.5)	2.5 (5.2)	---^b^ (0.4)

^a^ Values for *C_s_* complexes are given in parentheses; ^b^ CT energies are less than 0.1 kJ**^.^**mol^–1^.

**Table 3:** Binding energies (–ΔE, kJ**^.^**mol^–1^), N–C and S–S distances (R, Å), N–C–S and S–S–A angles. (<, ^o^), and spin-spin coupling constants ^a 1c^J(S–S) for *C_s_* HN(CH)SX:SCO complexes.

X =	–ΔE	R(N4–C1)	R(S3–S6)	<N4–C1–S3	<S3–S6–A7 ^b^	^1c^J(S–S)
F	15.3	3.004	3.348	95	169	5.7
Cl	14.7	3.051	3.489	96	166	3.8
NC	14.1	3.060	3.508	97	168	3.6
CCH	13.6	3.067	3.663	97	169	2.1
H	13.0	3.064	3.747	97	174	1.4
CN	13.2	3.075	3.628	97	169	2.3

^a 1t^J(N–C) = −0.3 Hz for all complexes; ^b^ A is the X atom directly bonded to S6.

**Table 4:** Binding energies (–ΔE, kJ**^.^**mol^–1^), N–C and S–S distances (R, Å), and N–C–S and S–S–A angles (<, ^o^) for molecules HN(CH)SX:SCO.

X =	–ΔE	R(N4–C1)	R(S3–S6)	<N4–C1–S3	<S3–S6–A7 ^a^
F	48.1	1.436	2.292	131	175
Cl	24.3	1.438	2.314	131	177
NC	–9.6	1.443	2.294	131	174
CCH	–65.1	1.536	2.803	137	175
H	–64.0	1.548	2.885	137	170
CN	–69.9	1.523	2.672	136	174

^a^ A is the X atom directly bonded to S6.

**Table 5:** Binding energies (–ΔE, kJ·mol^–1^), N–C, S–S, and S–A distances (R, Å), and N–C–S, S–S–A, and S–C–O angles (<, ^o^) for the HN(CH)SF:SCO and HN(CH)SCl:SCO complexes, transition structures, and molecules.

**X = F**	**–ΔE**	**R(N4–C1)**	**R(S3–S6)**	**R(S6–F7)**	**<N4–C1–S3**	**<S3–S6–F7**	**<S3–C1–O2**
complex	15.3	3.004	3.348	1.636	95	167	179
TS	–18.8	2.036	2.614	1.683	100	178	154
molecule	48.1	1.436	2.292	1.798	109	175	131
**X = Cl**	**–ΔE**	**R(N4–C1)**	**R(S3–S6)**	**R(S6–Cl7)**	**<N4–C1–S3**	**<S3–S6–Cl7**	**<S3–C1–O2**
complex	14.7	3.051	3.489	2.037	96	166	179
TS	–37.1	1.967	2.676	2.113	102	180	152
molecule	24.3	1.438	2.314	2.311	109	177	131

**Table 6:** Spin-spin coupling constants (Hz) for the HN(CH)SF:SCO and HN(CH)SCl:SCO complexes, transition structures, and molecules ^a^.

X =	Complex ^1t^J(N1–C4) ^a^	TS ^1t^J(N1–C4)	Molecule ^1^J(N–C)	Complex ^1c^J(S–S)	TS ^1c^J(S–S)	Molecule ^1c^J(S–S)
F	–0.3	17.9	–7.8	5.7	22.7	13.1
Cl	–0.3	21.0	–7.6	3.8	21.5	12.7

^a^ Data are given for those systems having bound molecules.

## Supplementary Materials

The following are available online, Table S1: Structures (Å), total energies (a.u.) and molecular graphs of HN(CH)SX:SCO complexes with C_1_ symmetry, Table S2: Structures (Å), total energies (a.u.) and molecular graphs of HN(CH)SX:SCO complexes with C_s_ symmetry, Table S3: Structures (Å), total energies (a.u.) and molecular graphs of HN(CH)SX:SCO molecules, Table S4: Structures (Å), total energies (a.u.) and molecular graphs of HN(CH)SX:SCO transition structures, Table S5: PSO, DSO, FC, and SD components of ^1t^J(N-C) and ^1c^J(S-S) for complexes, bound molecules, and transition structures with C_s_ symmetry.

## References

[1] Pimentel G.C., McClellan A.L. (1960). The Hydrogen Bond.

[2] Metrangolo P., Resnati G. (2008). Halogen Bonding. Fundamentals and Applications.

[3] Metrangolo P., Resnati G. (2015). Halogen Bonding I. Impact on Materials Chemistry and Life Sciences.

[4] (2017). Halogen Bonding in Supramolecular and Solid State Chemistry—Faraday Discussion.

[5] Minyaev R.M., Minkin V.I. (1998). Theoretical Study of O → X (S, Se, Te) Coordination in Organic Compounds. Can. J. Chem..

[6] Sanz P., Yáñez M., Mó O. (2003). Resonance-Assisted Intramolecular Chalcogen–Chalcogen Interactions?. Chem. Eur. J..

[7] Bleiholder C., Werz D.B., Köppel H., Gleiter R. (2006). Theoretical Investigations on Chalcogen−Chalcogen Interactions: What Makes These Nonbonded Interactions Bonding?. J. Am. Chem. Soc..

[8] Sánchez-Sanz G., Trujillo C., Alkorta I., Elguero J. (2012). Intermolecular Weak Interactions in HTeXH Dimers (X=O, S, Se, Te): Hydrogen Bonds, Chalcogen–Chalcogen Contacts and Chiral Discrimination. ChemPhysChem.

[9] Scheiner S.A. (2011). New Noncovalent Force: Comparison of P···N Interaction with Hydrogen and Halogen Bonds. J. Chem. Phys..

[10] Zahn S., Frank R., Hey-Hawkins E., Kirchner B. (2011). Pnicogen Bonds: A New Molecular Linker?. Chem. Eur. J..

[11] Del Bene J.E., Alkorta I., Elguero J., Scheiner S. (2015). The Pnicogen Bond in Review: Structures, Binding Energies, Bonding Properties, and Spin−Spin Coupling Constants of Complexes Stabilized by Pnicogen Bonds. Noncovalent Forces; Challenges and Advances in Computational Chemistry and Physics.

[12] Alkorta I., Rozas I., Elguero J. (2001). Molecular Complexes between Silicon Derivatives and Electron-Rich Groups. J. Phys. Chem. A.

[13] Bauzá A., Mooibroek T.J., Frontera A. (2013). Tetrel-Bonding Interaction: Rediscovered Supramolecular Force?. Angew. Chem. Int. Ed..

[14] Grabowski S.J. (2014). Tetrel Bond-σ-Hole Bond as a Preliminary Stage of the SN_2_ Reaction. Phys. Chem. Chem. Phys..

[15] Murray J.S., Lane P., Politzer P. (2009). Expansion of the σ-Hole Concept. J. Mol. Model..

[16] Politzer P., Murray J.S., Clark T. (2013). Halogen Bonding and Other σ-Hole Interactions: A Perspective. Phys. Chem. Chem. Phys..

[17] Desiraju G.R., Ho P.S., Kloo L., Legon A.C., Marquardt R., Metrangolo P., Politzer P., Resnati G., Rissanen K. (2013). Definition of the Halogen Bond (IUPAC Recommendations 2013). Pure Appl. Chem..

[18] Del Bene J.E., Alkorta I., Elguero J. (2017). Carbenes as Electron-Pair Donors to CO_2_ for C···C Tetrel Bonds and C−C Covalent Bonds. J. Phys. Chem. A.

[19] Alkorta I., Elguero J., Del Bene J.E. (2017). Azines as Electron-Pair Donors to CO_2_ for N···C Tetrel Bonds. J. Phys. Chem. A.

[20] Del Bene J.E., Alkorta I., Elguero J. (2017). Carbon−Carbon Bonding between Nitrogen Heterocyclic Carbenes and CO_2_. J. Phys. Chem. A.

[21] Del Bene J.E., Alkorta I., Elguero J. (2018). Complexes of CO_2_ with the Azoles: Tetrel Bonds, Hydrogen Bonds and Other Secondary Interactions. Molecules.

[22] Del Bene J.E., Alkorta I., Elguero J. (2019). Potential Energy Surfaces of HN(CH)SX:CO_2_ for X=F, Cl, NC, CN, CCH, and H: N···C Tetrel Bonds and O···S Chalcogen Bonds. J. Phys. Chem. A.

[23] Alkorta I., Elguero J., Del Bene J.E. (2018). Complexes of O=C=S with Nitrogen Bases: Chalcogen Bonds, Tetrel Bonds, and Other Secondary Interactions. ChemPhysChem.

[24] Del Bene J.E., Alkorta I., Elguero J. (2019). Exploring N···C Tetrel and O···S Chalcogen Bonds in HN(CH)SX:OCS Systems, for X=F, NC, Cl, CN, CCH, and H. Chem. Phys. Lett..

[25] Elguero J., Katritzky A.R., Marzin C., Linda P. (1976). The Tautomerism of Heterocycles.

[26] Salina E., Ryabova O., Kaprelyants A., Makarov V. (2014). New 2-Thiopyridines as Potential Candidates for Killing both Actively Growing and Dormant Mycobacterium tuberculosis Cells. Antimicrob. Agents Chemother..

[27] Pople J.A., Binkley J.S., Seeger R. (1976). Theoretical Models Incorporating Electron Correlation. Int. J. Quantum Chem..

[28] Krishnan R., Pople J.A. (1978). Approximate Fourth-Order Perturbation Theory of the Electron Correlation Energy. Int. J. Quantum Chem..

[29] Bartlett R.J., Silver D.M. (1975). Many-Body Perturbation Theory Applied to Electron Pair Correlation Energies. I. Closed-Shell First Row Diatomic Hydrides. J. Chem. Phys..

[30] Bartlett R.J., Purvis G.D. (1978). Many-Body Perturbation Theory, Coupled-Pair Many-Electron Theory, and the Importance of Quadruple Excitations for the Correlation Problem. Int. J. Quantum Chem..

[31] Del Bene J.E. (1993). Proton Affinities of Ammonia, Water, and Hydrogen Fluoride and Their Anions: A Quest for the Basis-Set Limit Using the Dunning Augmented Correlation-Consistent Basis Sets. J. Phys. Chem..

[32] Dunning T.H. (1989). Gaussian Basis Sets for Use in Correlated Molecular Calculations. I. The Atoms Boron through Neon and Hydrogen. J. Chem. Phys..

[33] Woon D.E., Dunning T.H. (1995). Gaussian basis sets for use in correlated molecular calculations. V. Core-valence basis sets for boron through neon. J. Chem. Phys..

[34] Trucks G.W., Schlegel H.B., Scuseria G.E., Robb M.A., Cheeseman J.R., Scalmani G., Barone V., Petersson G.A., Nakatsuji H., Li X. (2016). Gaussian 16, Revision A. 03.

[35] Reed A.E., Curtiss L.A., Weinhold F. (1988). Intermolecular Interactions from a Natural Bond Orbital, Donor–Acceptor Viewpoint. Chem. Rev..

[36] Glendening E.D., Badenhoop J.K., Reed A.E., Carpenter J.E., Bohmann J.A., Morales C.M., Landis C.R., Weinhold F. (2013). NBO 6.0.

[37] Bader R.F.W. (1991). A Quantum Theory of Molecular Structure and its Applications. Chem. Rev..

[38] Bader R.F.W. (1990). Atoms in Molecules. A Quantum Theory.

[39] Popelier P.L.A. (2000). Atoms in Molecules. An Introduction.

[40] Matta C.F., Boyd R.J. (2007). The Quantum Theory of Atoms in Molecules: From Solid State to DNA and Drug Design.

[41] Keith T.A. (2017). AIMAll (Version 17.11.14B).

[42] Perera S.A., Nooijen M., Bartlett R.J. (1996). Electron Correlation Effects on the Theoretical Calculation of NuclearMagnetic Resonance Spin-Spin Coupling Constants. J. Chem. Phys..

[43] Perera S.A., Sekino H., Bartlett R.J. (1994). Coupled-Cluster Calculations of Indirect Nuclear Coupling Constants: The Importance of Non-Fermi Contact Contributions. J. Chem. Phys..

[44] Schäfer A., Horn H., Ahlrichs R. (1992). Fully Optimized Contracted Gaussian Basis Sets for Atoms Li to Kr. J. Chem. Phys..

[45] Stanton J.F., Gauss J., Watts J.D., Nooijen J.M., Oliphant N., Perera S.A., Szalay P.G., Lauderdale W.J., Gwaltney S.R., Beck S. (1991). ACES II.

