# Supplementary material for: N…C and S…S Interactions in Complexes, Molecules, and Transition Structures HN(CH)SX:SCO, for X = F, Cl, NC, CCH, H, and CN
Source: Molecules. 2019 Sep 5;24(18):3232. doi: 10.3390/molecules24183232 (PMC6767182; doi:10.3390/molecules24183232)
Supplement: Supplementary file 1 [file molecules-24-03232-s001.zip › molecules-583848-SI.pdf]

Supporting Information for

N···C and S···S Interactions in Complexes, Molecules, and Transition Structures HN(CH)SX:SCO, for X = F, Cl, NC, CCH, H, and CN

Janet E. Del Bene,[a,*] Ibon Alkorta,[b,*] José Elguero[b]

Pgs. S2-S3: Table S1. Structures (Å), total energies (a.u.) and molecular graphs of HN(CH)SX:SCO complexes with C_1 symmetry

Pgs. S4-S5: Table S2. Structures (Å), total energies (a.u.) and molecular graphs of HN(CH)SX:SCO complexes with C_s symmetry

Pgs. S6-S7: Table S3. Structures (Å), total energies (a.u.) and molecular graphs of HN(CH)SX:SCO molecules

Pgs. S8-S9: Table S4. Structures (Å), total energies (a.u.) and molecular graphs of HN(CH)SX:SCO transition structures

Pg. S10: Table S5. PSO, DSO, FC, and SD components of ^{1t}J(N-C) and ^{1c}J(S-S) for complexes, bound molecules, and transition structures with C_s symmetry

Table S1. Structures (Å), total energies (a.u.) and molecular graphs of HN(CH)SX:SCO complexes with C_1 symmetry

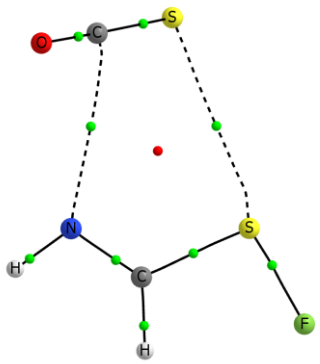	hnchsf_sco_complex<br>MP2= -1102.18853592 NIM= 0<br>C,-0.6737495662,-2.2012722223,0.1645530725<br>O,0.0532609366,-2.9268096337,0.7169785976<br>S,-1.6766472384,-1.2436895447,-0.5714897163<br>N,1.6918437011,-0.4329296105,-0.3938703477<br>C,1.560761095,0.8154417164,-0.1761502041<br>S,-0.0118818309,1.5304951395,0.1266985267<br>F,0.5368481926,3.0523713662,0.3768651143<br>H,2.3488139606,1.5723381045,-0.1463712087<br>H,2.6733607497,-0.6558383154,-0.5522138342
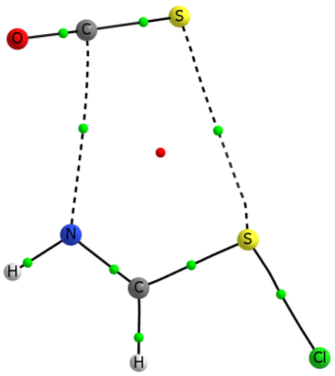	hnchscl_sco_complex<br>MP2= -1462.18814858 NIM= 0<br>C,-0.6923592381,-2.2915930954,0.1533796536<br>O,0.0737255525,-2.9619502662,0.7243677845<br>S,-1.7412821452,-1.4070577005,-0.6065270613<br>N,1.6780357802,-0.4379893331,-0.3952330875<br>C,1.5873541237,0.8123939283,-0.1800105286<br>S,-0.0101869908,1.5095375729,0.1114305684<br>Cl,0.5607083959,3.4382425066,0.4376474273<br>H,2.3950202421,1.5463124995,-0.1495910041<br>H,2.6515942799,-0.6977891119,-0.5504637522
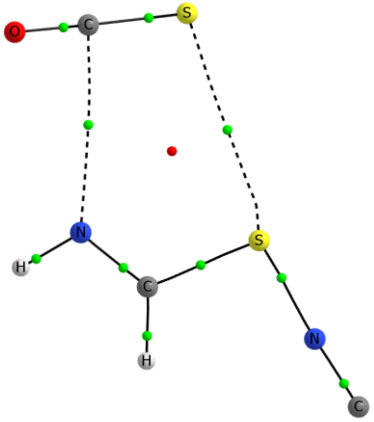	hnchsnc_sco_complex<br>MP2= -1095.10126112 NIM= 0<br>C,-0.7005348145,-2.3694582265,0.0511743103<br>O,0.1152278371,-3.1733114488,0.2738593347<br>S,-1.816932929,-1.3081469626,-0.2469797248<br>N,1.6511863137,-0.4221899418,-0.1987426584<br>C,1.6149827897,0.8425103602,-0.1055645497<br>S,0.0473990238,1.6186707028,0.191208042<br>N,0.6066224733,3.1842652828,0.2630250685<br>H,2.4383166599,1.5547617205,-0.1846916768<br>H,2.6044911887,-0.7393999663,-0.3720943088<br>C,0.9418514574,4.3224054797,0.3238061631

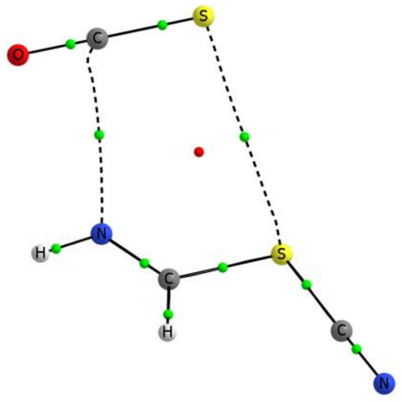	hnchscn:sco cis<br>MP2/aug'-cc-pVTZ= -1095.15217600 NIM= 0<br>C,2.4199929934,0.1969289269,0.4568714623<br>O,2.8603698776,1.2799265172,0.4884904382<br>S,1.8324594079,-1.2540436015,0.4290613475<br>N,0.144804519,1.6174971991,-0.9769258203<br>C,-0.9724404353,1.2212324885,-0.5268584171<br>S,-1.5449181791,-0.3756103009,-1.070868781<br>C,-3.0034598136,-0.3909863304,-0.2102787847<br>H,-1.6442847732,1.7321392253,0.1634327174<br>H,0.3775133739,2.5370080082,-0.60115141<br>N,-4.0199054856,-0.4198807296,0.3815658843
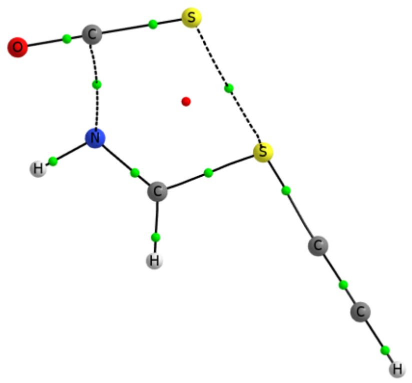	hnchscch_sco_complex<br>MP2= -1079.05835662 NIM= 0<br>C,2.4226930363,0.2019940394,0.4582025033<br>O,2.8939687854,1.2722932309,0.4866111315<br>S,1.7957537097,-1.2321141238,0.4357598273<br>N,0.1625669426,1.6242259562,-0.9810356403<br>C,-0.9600100278,1.2219914518,-0.5392486697<br>S,-1.5576315051,-0.3566654326,-1.077349409<br>C,-3.0060548403,-0.3959680368,-0.2084111488<br>H,-1.6279974044,1.7364065759,0.153460286<br>H,0.3867981071,2.5411537906,-0.5943407163<br>C,-4.0599553185,-0.4691060487,0.3996904726<br>H,-4.9790334849,-0.5330784029,0.9296943634
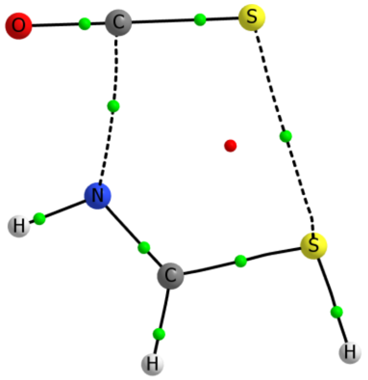	hnchsh_sco_complex<br>MP2= -1003.07649272 NIM= 0<br>C,1.8401868177,0.4048395797,0.2528756209<br>O,2.1563399266,1.5148450983,0.0644995737<br>S,1.4337424868,-1.0839670627,0.5161671165<br>N,-0.8023316074,1.2917375902,-0.8981857995<br>C,-1.8470145358,0.8158146162,-0.3419338303<br>S,-2.221844445,-0.8899930558,-0.5384795195<br>H,-3.3488713611,-0.8526838351,0.1801621361<br>H,-2.55393354,1.3792434868,0.2675654599<br>H,-0.7187327417,2.2867165823,-0.6910147578

Table S2. Structures (Å), total energies (a.u.) and molecular graphs of HN(CH)SX:SCO complexes with C_s symmetry

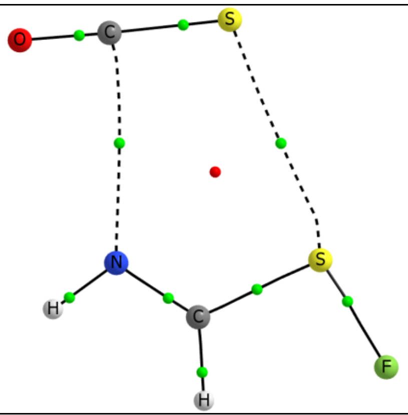	hnchsf_sco_complex<br>MP2= -1102.18845511 NIM= 1<br>C,1.9199943898,-0.611200373,0.<br>O,2.7901716149,0.165112193,0.<br>S,0.7763398217,-1.6866408715,0.<br>N,0.0484660751,1.7389333927,0.<br>C,-1.2234788261,1.6671397561,0.<br>S,-2.0436047254,0.1183862475,0.<br>F,-3.5642190488,0.7224074973,0.<br>H,-1.9382137244,2.4941736145,0.<br>H,0.3418534232,2.7146735433,0.
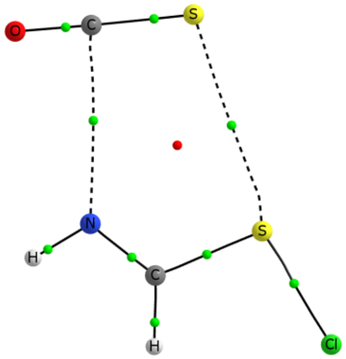	hnchscl_sco_complex<br>MP2= -1462.18813028 NIM= 1<br>C,-0.0411475472,-2.8511648733,0.<br>O,0.9641324133,-3.4439750339,0.<br>S,-1.4083573103,-2.0825083712,0.<br>N,1.7319688777,-0.3678079423,0.<br>C,1.3805519378,0.8546096287,0.<br>S,-0.3433334243,1.2396187936,0.<br>Cl,-0.1986435192,3.2716152358,0.<br>H,2.0244835791,1.7362810425,0.<br>H,2.7487241231,-0.4423339499,0.
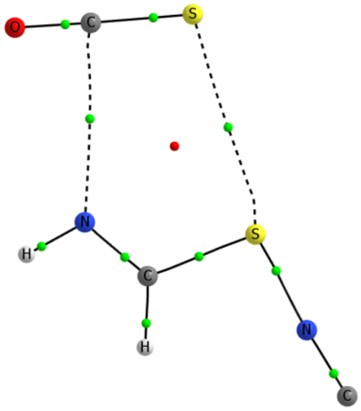	hnchsnc_sco_complex<br>MP2= -1095.10125561 NIM= 1<br>C,2.7909659225,0.0351406968,0.<br>O,3.3326268544,1.0685203331,0.<br>S,2.089021074,-1.367932566,0.<br>N,0.2274102038,1.7057841401,0.<br>C,-0.9786362191,1.3123531886,0.<br>S,-1.2923577592,-0.4337191999,0.<br>N,-2.9537375014,-0.3419887779,0.<br>H,-1.8905217838,1.9123274895,0.<br>H,0.2702664322,2.7244027264,0.<br>C,-4.1417541084,-0.3445680625,0.

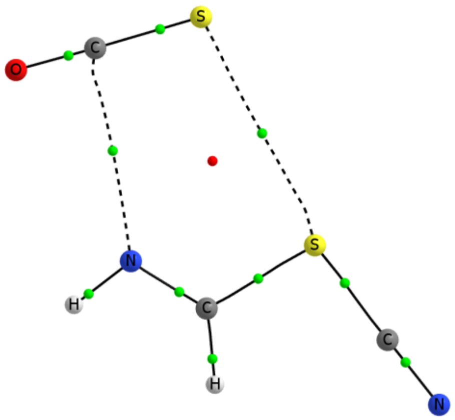	hnchscn_sco_complex<br>MP2= -1095.15209109 NIM= 1<br>C,2.8363338618,0.0520420714,0.<br>O,3.3394141043,1.1054432049,0.<br>S,2.185568671,-1.3745237355,0.<br>N,0.2199812178,1.667546745,0.<br>C,-0.9930093614,1.2989600964,0.<br>S,-1.3237014804,-0.454225243,0.<br>C,-3.0148706138,-0.3446491173,0.<br>H,-1.8872889443,1.9228005532,0.<br>H,0.2810039429,2.6858049148,0.<br>N,-4.1901482829,-0.2888795217,0.
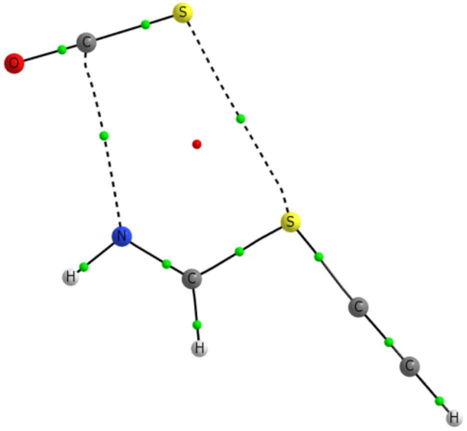	hnchscch_sco_complex<br>MP2= -1079.05817922 NIM= 1<br>C,2.8499161047,0.0588550591,0.<br>O,3.3553554619,1.1115709409,0.<br>S,2.1987189963,-1.3668386547,0.<br>N,0.2359967458,1.6635413005,0.<br>C,-0.9804692784,1.2939493801,0.<br>S,-1.3457418092,-0.4419173124,0.<br>C,-3.0329496815,-0.3409959917,0.<br>H,-1.8681096586,1.9286793753,0.<br>H,0.2922737051,2.6818479305,0.<br>C,-4.2517074712,-0.3183720594,0.<br>H,-5.3144243049,-0.2994035581,0.
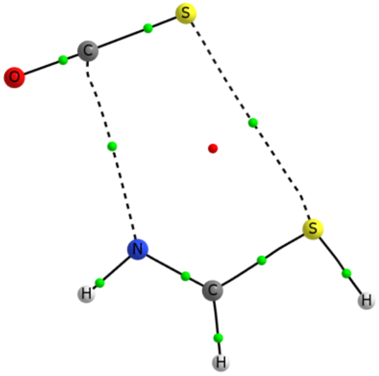	hnchsh_sco_complex<br>MP2= -1003.07630771 NIM= 1<br>C,1.7320890987,-0.646017749,0.<br>O,2.3572953422,0.340510009,0.<br>S,0.9205334934,-1.9865131691,0.<br>N,-0.6716846835,1.2532005561,0.<br>C,-1.9322723803,1.0584698426,0.<br>S,-2.5549533518,-0.5865501108,0.<br>H,-3.8425676262,-0.2250146688,0.<br>H,-2.6926039885,1.8398614301,0.<br>H,-0.4762286941,2.2538528698,0.

Table S3. Structures (Å), total energies (a.u.) and molecular graphs of HN(CH)SX:SCO molecules

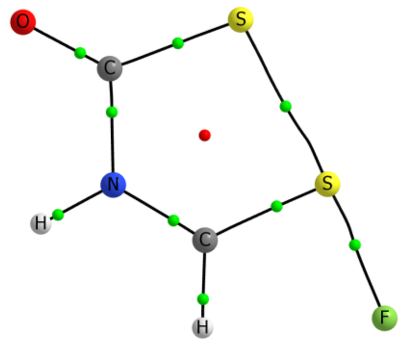	hnchsf_sco_molecule<br>MP2= -1102.20092699 NIM= 0<br>C,0.1063964564,1.350975688,0.<br>O,0.231183774,2.5570439469,0.<br>S,-1.3001833888,0.3511348492,0.<br>N,1.273162898,0.5132232652,0.<br>C,1.2034918558,-0.8048855171,0.<br>S,-0.2173990149,-1.6692778057,0.<br>F,0.7599321918,-3.1780207589,0.<br>H,2.1165081114,-1.3843848435,0.<br>H,2.1723601163,0.984028176,0.
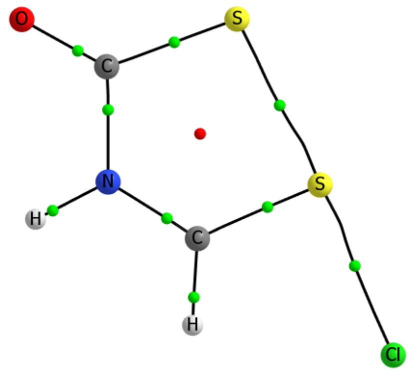	hnchscl_sco_molecule<br>MP2= -1462.1917678 NIM= 0<br>C,0.1161868026,1.4166332426,0.<br>O,0.2716247517,2.6174942145,0.<br>S,-1.3259788568,0.4641802164,0.<br>N,1.2615645574,0.5475908076,0.<br>C,1.1688783233,-0.7678480425,0.<br>S,-0.2783576407,-1.5987588241,0.<br>Cl,0.8865985927,-3.5942712801,0.<br>H,2.072658111,-1.3624128292,0.<br>H,2.1722783589,0.9972294949,0.
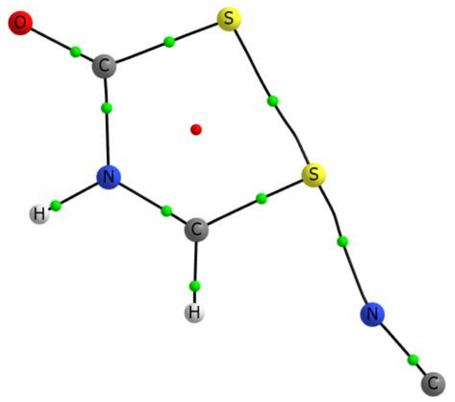	hnchsnc_sco_molecule<br>MP2= -1095.0922502 NIM= 0<br>C,0.0787267131,1.3895684502,0.<br>O,0.1817233793,2.5945067875,0.<br>S,-1.310846511,0.3648462023,0.<br>N,1.2684353517,0.5725842376,0.<br>C,1.2381702521,-0.7414880446,0.<br>S,-0.1791902297,-1.6307590838,0.<br>N,0.9488179833,-3.2300599251,0.<br>H,2.1675940612,-1.2945402088,0.<br>H,2.1571375029,1.0651968332,0.<br>C,1.2948844973,-4.3700182485,0.

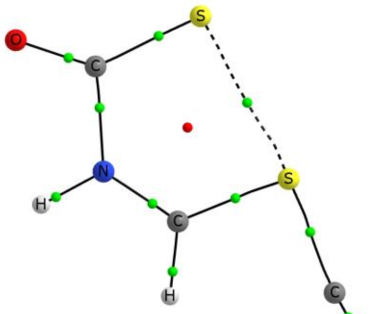	hnchscn_sco_molecule<br>MP2= -1095.1204361 NIM= 0<br>C,0.009571444,1.4320832404,0.<br>O,0.3186794481,2.6047735328,0.<br>S,-1.4221442279,0.5529594587,0.<br>N,1.220090029,0.5082739778,0.<br>C,1.2407023746,-0.7857947808,0.<br>S,-0.1442472918,-1.7938030826,0.<br>C,0.8717807877,-3.2461208971,0.<br>H,2.2063599189,-1.2772290275,0.<br>H,2.1099169207,1.004490613,0.<br>N,1.4347435968,-4.2797960346,0.
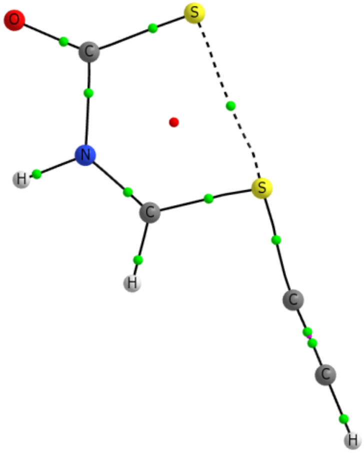	hnchscch_sco_molecule<br>MP2= -1079.02817534 NIM= 0<br>C,-0.012510738,1.4593382669,0.<br>O,0.3317865051,2.625147803,0.<br>S,-1.454385114,0.6115119459,0.<br>N,1.2028977269,0.5196595892,0.<br>C,1.2475925176,-0.7746995867,0.<br>S,-0.0920458612,-1.83817435,0.<br>C,0.889233472,-3.2642518021,0.<br>H,2.2237948352,-1.245901089,0.<br>H,2.0872569947,1.0248105726,0.<br>C,1.4394293875,-4.3525142537,0.<br>H,1.9324032742,-5.2950900959,0.
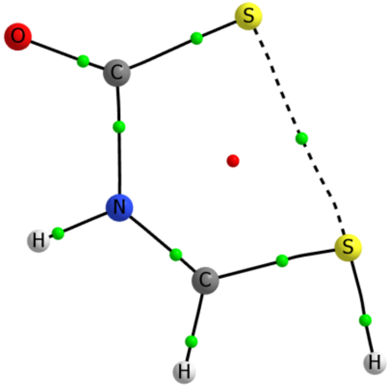	hnchsh_sco_molecule<br>MP2= -1003.04696789 NIM= 0<br>C,-1.35774839,0.17159005,0.<br>O,-2.45793305,0.68937447,0.<br>S,-0.73573469,-1.37730904,0.<br>N,-0.25179916,1.25422975,0.<br>C,1.03884101,1.14390843,0.<br>S,1.94147986,-0.30217892,0.<br>H,3.08959592,0.42288161,0.<br>H,1.60315247,2.06829798,0.<br>H,-0.64516836,2.19303285,0.

Table S4. Structures (Å), total energies (a.u.) and molecular graphs of HN(CH)SX:SCO transition structures

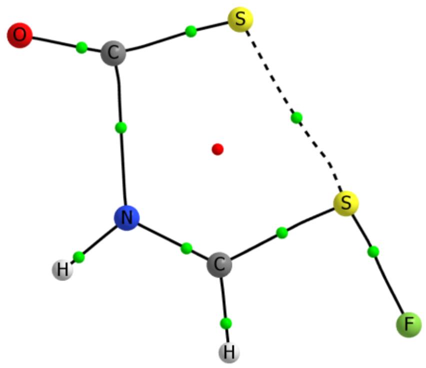	hnchsf_sco TS<br>MP2= -1102.17544713 NIM= 1<br>C,0.0608423,-1.94405723,0.<br>O,0.66399223,-2.9498708,0.<br>S,-1.29320703,-1.05383991,0.<br>N,1.4626025,-0.46807969,0.<br>C,1.13380193,0.76849725,0.<br>S,-0.46972481,1.42721692,0.<br>F,0.12879968,3.00045113,0.<br>H,1.86078351,1.58010479,0.<br>H,2.46890802,-0.6293129,0.
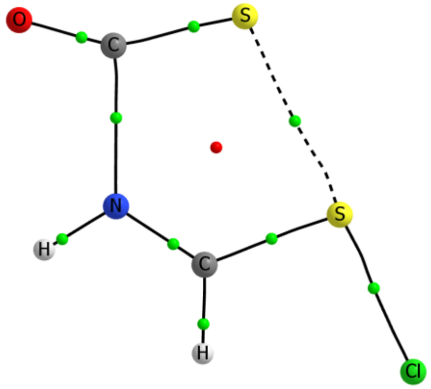	hnchscl_sco TS<br>MP2= -1462.16840470 NIM= 1<br>C,0.12700093,-2.31974361,0.<br>O,0.76871742,-3.30552958,0.<br>S,-1.29818323,-1.53716223,0.<br>N,1.41858786,-0.83596943,0.<br>C,1.09844807,0.40374586,0.<br>S,-0.53089991,1.02677632,0.<br>Cl,0.0922804,3.04548007,0.<br>H,1.84145033,1.19805858,0.<br>H,2.42256478,-1.01303605,0.
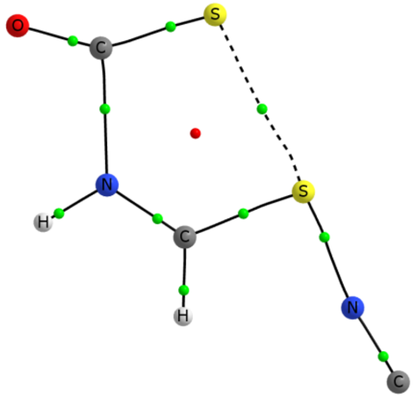	hnchsnc_sco TS<br>MP2= -1095.07701872 NIM= 1<br>C,0.1730483003,-2.139196054,0.<br>O,0.8135815925,-3.1311381818,0.<br>S,-1.2973618812,-1.4251132347,0.<br>N,1.3824372683,-0.7047555131,0.<br>C,1.0760088642,0.5356072929,0.<br>S,-0.5508564413,1.1694720215,0.<br>N,0.0251400337,2.7913531271,0.<br>H,1.8296451234,1.3189296148,0.<br>H,2.3799644125,-0.9148899031,0.<br>C,0.2509527276,3.9584263503,0.

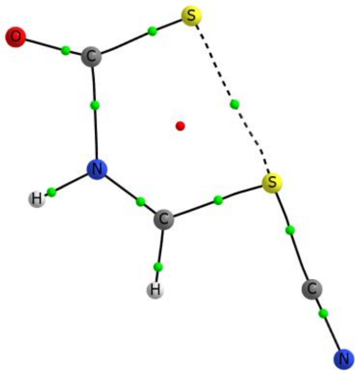	hnchscn_sco TS<br>MP2= -1095.11902037 NIM= 1<br>C,0.2462561989,-2.0721796613,0.<br>O,0.921117524,-3.0585386876,0.<br>S,-1.3119178862,-1.5367062575,0.<br>N,1.2994690249,-0.7129358278,0.<br>C,1.0291081911,0.5392586882,0.<br>S,-0.5808995851,1.2053291466,0.<br>C,0.0173114313,2.8324688387,0.<br>H,1.8315420202,1.2703846537,0.<br>H,2.2858949426,-0.9708087972,0.<br>N,0.3446781382,3.9624234244,0.
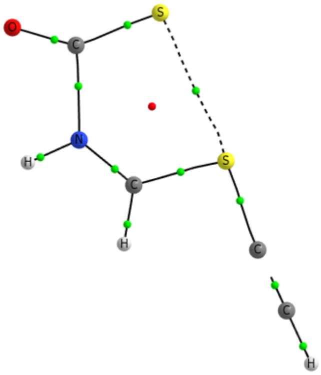	hnchscch_sco TS<br>MP2= -1079.02704899 NIM= 1<br>C,0.2636861431,-2.1200605692,0.<br>O,0.9743241644,-3.0839767314,0.<br>S,-1.3098836561,-1.6410426669,0.<br>N,1.2862433521,-0.7345798379,0.<br>C,1.011635139,0.5199196695,0.<br>S,-0.5809514086,1.20527495,0.<br>C,-0.0335379315,2.8287437433,0.<br>H,1.8150905986,1.25074617,0.<br>H,2.2739554527,-0.9853240094,0.<br>C,0.2379814717,4.0170156219,0.<br>H,0.4833905144,5.05189587,0.
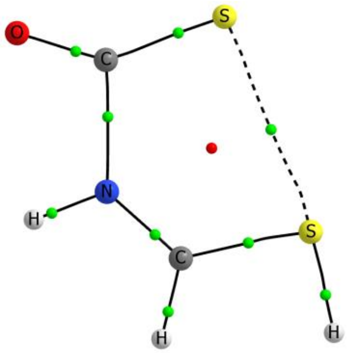	hnchsh_sco TS<br>MP2= -1003.04605350 NIM= 1<br>C,0.16321793,-1.43822369,0.<br>O,0.80530004,-2.45067152,0.<br>S,-1.37485005,-0.85768913,0.<br>N,1.29186504,-0.13777209,0.<br>C,1.13951492,1.13957109,0.<br>S,-0.36602918,1.97742089,0.<br>H,0.27636136,3.16306297,0.<br>H,2.02574837,1.76530447,0.<br>H,2.25010531,-0.4823831,0.

Table S5. PSO, DSO, FC, and SD components of ^{1t}J(N-C) and ^{1c}J(S-S) (Hz) for complexes, bound molecules, and transition structures with C_s symmetry

Complexes					
X =	PSO	DSO	FC	SD	^{1t}J(N-C)
F	0.0	0.0	-0.3	0.0	-0.3
Cl	0.0	0.0	-0.3	0.0	-0.3
NC	0.0	0.0	-0.3	0.0	-0.3
CCH	0.0	0.0	-0.3	0.0	-0.3
H	0.0	0.0	-0.3	0.0	-0.3
CN	0.0	0.0	-0.3	0.0	-0.3
					^{1c}J(S-S)
F	0.1	0.0	5.7	0.0	5.7
Cl	0.0	0.0	3.8	0.0	3.8
NC	0.0	0.0	3.6	0.0	3.6
CCH	0.0	0.0	2.1	0.0	2.1
H	0.0	0.0	1.5	0.0	1.4
CN	0.0	0.0	2.3	0.0	2.3

Molecules					
	PSO	DSO	FC	SD	^{1}J(N-C)
X = F	1.7	-0.2	-9.0	-0.3	-7.8
Cl	1.6	-0.2	-8.8	-0.3	-7.6
TS					^{1t}J(N-C)
X = F	0.2	-0.1	17.9	-0.1	17.9
Cl	0.2	-0.1	21.1	-0.2	21.0

Molecules	PSO	DSO	FC	SD	^{1c}J(S-S)
X = F	-1.3	0.0	13.5	0.9	13.1
Cl	-0.5	0.0	11.7	1.5	12.7
TS					
F	0.0	0.0	22.6	0.1	22.7
Cl	0.0	0.0	21.5	0.0	21.5
